# The Influence of Maternal Folate Status on Gestational Diabetes Mellitus: A Systematic Review and Meta-Analysis

**DOI:** 10.3390/nu15122766

**Published:** 2023-06-16

**Authors:** Ruhan Xu, Shenhao Liu, Zhiqi Zhong, Yifei Guo, Tianqi Xia, Yanyan Chen, Lingling Ding

**Affiliations:** 1 School of Medicine, Jiangsu University, Zhenjiang 212013, China; xuruhan1119@163.com (R.X.); lsh010224@163.com (S.L.); gluacid@163.com (Z.Z.); 18862790867@163.com (Y.G.); xiatodd@163.com (T.X.); yanyanchen@ujs.edu.cn (Y.C.); 2 Department of Pharmacology, School of Medicine, Jiangsu University, Zhenjiang 212013, China; 3 Department of Physiology, School of Medicine, Jiangsu University, Zhenjiang 212013, China; 4 Key Laboratory of Laboratory Medicine of Jiangsu Province, School of Medicine, Jiangsu University, Zhenjiang 212013, China

**Keywords:** gestational diabetes mellitus, GDM risk, folate, different stage of pregnancy, meta-analysis

## Abstract

Maternal folate has been shown to relate to the risk of gestational diabetes mellitus (GDM). However, the existing studies have yielded inconsistent conclusions. The purpose of this study was to systematically review the association between maternal folate status and the risk of GDM. Observational studies up to 31 October 2022 were included. Study characteristics, the means and standard deviations (SDs) of folate levels (serum/red blood cell (RBC)), the odds ratios (ORs) with 95% confidence intervals (CIs) and the time for folate measurement were extracted. Compared with the non-GDM group, serum and RBC folate levels in women with GDM were significantly higher. Our subgroup analysis demonstrated that serum folate levels in the GDM group were significantly higher than in the non-GDM group only in the second trimester. RBC folate levels in the GDM group were significantly higher than in the non-GDM group in the first and second trimesters. Taking serum/RBC folate levels as continuous variables, the adjusted odds ratios of GDM risk showed that increased serum folate concentration rather than RBC folate elevated the risk of GDM. In the descriptive analysis, five studies reported high serum folate levels increased GDM risk, whereas the other five showed no association between serum folate levels and GDM risk. Moreover, the rest three studies pointed out high RBC folate levels increased GDM risk. Altogether we found that the risk of GDM is associated with high serum/plasma and RBC folate levels. Future studies should determine the recommended folic acid cutoff balancing the risk for GDM and fetal malformations.

## 1. Introduction

With the rising of obesity, GDM has become a common complication of pregnancy [1]. According to “Classification and Diagnosis of Diabetes: Standards of Care in Diabetes—2023” from the American diabetes association, GDM is classified as diabetes diagnosed in the second or third trimester of pregnancy, not overt diabetes before gestation [2]. In some countries, including developing countries, the prevalence of GDM has increased by more than 30% in the past 10 to 20 years [3]. Indeed, GDM has affected a large number of pregnant women around the world and imposed an economic and health burden on society. Notably, GDM has multiple adverse implications for the health of current and future generations from genetic and environmental perspectives [4]. The main risk factors of GDM include overweight, maternal obesity, late childbearing age, previous GDM history, and family history of type 2 diabetes [1]. GDM can increase the risk of adverse pregnancy outcomes for pregnant women, such as perinatal mortality and caesarean section rates [5]. Besides, GDM also increases the risk of mothers with diabetes and other cardiovascular diseases [6,7,8]. Altogether, GDM poses a huge threat to the health of pregnant women and their fetuses. Thus, identifying the potential risk factors of GDM is important for preventing GDM and improving the health of pregnant women and newborns.

Folate is a water-soluble vitamin which plays a vital role in DNA methylation, nucleic acids and protein synthesis, making it a necessary nutrient for early pregnancy. As folate cannot be synthesized by the human body, it must be obtained from food or supplements. The demand for folic acid increases during pregnancy to support normal fetal development [9]. In the daily diet, folic acid mainly comes from animal liver, eggs, beans, yeast, green leafy vegetables, fruit, and nuts. However, as folic acid in natural food is easy to decompose after cooking and processing, the amount of folic acid obtained from food alone is insufficient for pregnant women due to the loss of folic acid. Therefore, folic acid supplementation or folic acid-containing multivitamins are recommended in clinics to prevent neural tube defects (NTDs) [10,11,12].

Notably, several studies have indicated that high folate concentrations may lead to immune imbalances and exacerbate vitamin B12 deficiency, which further trigger insulin resistance via inflammation and mitochondrial stress, respectively, thus promoting the development of GDM [13,14,15,16,17,18]. Therefore, it is essential to evaluate the effect of folate on GDM.

Surprisingly, studies have found a link between folate and GDM, but their conclusions are inconsistent. For example, Zhao et al. [19] found that folic acid supplementation before pregnancy can reduce the risk of GDM by 27% (OR 0.73, 95% CI 0.69, 0.79), while another Chinese cohort study [20] observed that folic acid supplementation before pregnancy can increase the risk of GDM (OR 1.72, 95% CI 1.17, 2.53). The following reasons might explain above mentioned differences. On the one hand, the absorption and metabolism of folate may vary from person to person, leading to different levels of serum and RBC folate [21]. On the other hand, due to personal compliance, it is not necessarily accurate to evaluate the specific intake of folic acid from supplements or a natural diet. Serum/plasma folate and RBC folate are the objective indicators of circulating folate levels, which are more reliable. Therefore, we believe it is more valuable to evaluate the relationship between serum/plasma and RBC folate concentration and the risk of GDM. To this end, we comprehensively evaluated the relationship between serum/plasma folate levels, RBC folate levels, and GDM risk in the current study.

To our knowledge, four meta-analysis articles have been published on the association between maternal folate levels and GDM [22,23,24,25]. However, their results differ, and the number of articles included is also limited. Therefore, this paper systematically searched the relevant literatures till 31 October 2022 to comprehensively evaluate the relationship between maternal folate status and GDM risk.

## 2. Materials and Methods

### 2.1. Search Strategy

This systematic review was developed following the Preferred Reporting Items for Systematic Reviews and Meta-analyses (PRISMA) 2020 statement [26]. We searched Pubmed, Embase, The Cochrane Library, and Web of Science databases to collect literature on the relationship between folate and GDM. All databases were screened from inception to 31 October 2022. The search was conducted independently by two researchers, and the final results were determined after a mutual discussion of the inconsistencies. Taking Pubmed as an example, for GDM, we used keywords such as diabetes, gestational diabetes, pregnancy-induced, pregnancy-induced diabetes, gestational diabetes, gestational diabetes mellitus, etc. For folate, keywords included vitamin M, B9, B9, and pteroylglutamic acid. The specific retrieval strategy was: ((“Diabetes, Gestational” [Mesh]) OR ((((((Diabetes, Pregnancy-Induced) OR (Diabetes, Pregnancy Induced)) OR (Pregnancy-Induced Diabetes)) OR (Gestational Diabetes)) OR (Diabetes Mellitus, Gestational)) OR (Gestational Diabetes Mellitus))) AND ((“Folic Acid” [Mesh]) OR ((((((((((((((Vitamin M) OR (Vitamin B9)) OR (B9, Vitamin)) OR (Pteroylglutamic Acid)) OR (Folic Acid, Monopotassium Salt)) OR (Folic Acid, Monosodium Salt)) OR (Folic Acid, Potassium Salt)) OR (Folic Acid, (DL)-Isomer)) OR (Folvite)) OR (Folacin)) OR (Folate)) OR (Folic Acid, (D)-Isomer)) OR (Folic Acid, Calcium Salt (1:1))) OR (Folic Acid, Sodium Salt))). The search strategy is listed in the Supplementary Material.

### 2.2. Inclusion and Exclusion Criteria

Inclusion criteria for the review were as follows: (1) Including cross-sectional study, case-control study, and cohort study; (2) GDM patients were diagnosed during pregnancy; (3) Two groups were studied, including one group of GDM group and the control group. The folate levels of GDM patients and non-GDM patients were compared; (4) The evaluation method of folate level was to measure serum or RBC concentration; (5) The study reported the effect estimate relative risk (RR), odds ratio (OR), and corresponding 95% confidence interval (CI), or could be converted into OR, RR, and 95% confidence interval; (6) For studies that reported a duplicate or overlapping data, studies with large sample size should be included.

The following studies were excluded: (1) Participants with multiple pregnancies or pregestational diabetes (type 1 or type 2 diabetes); (2) Review articles, non-English articles, case series, case reports, and conference papers; (3) The article did not provide the full text or the information provided was insufficient; (4) The study was performed in cellular and animal level, not in human.

### 2.3. Data Collection and Quality Assessment

Two independent researchers reviewed titles and abstracts for study selection, and studies that met the inclusion criteria were retrieved for full-text assessment. We extracted the following data from each selected study: first author, year, country, study design, sample size, number of GDM, age, test for GDM, GDM criteria, the period for GDM assessment, quality score, folate levels of GDM and Non-GDM, the corresponding indicators of outcome effect and correction for covariates. The methodological quality of the enrolled cohort/case-control studies was assessed by using the Newcastle-Ottawa Scale (NOS) [27], and cross-sectional studies using Agency for Healthcare Research and Quality (AHRQ) [28]. The NOS scale was scored based on three aspects of study object selection, including comparability, exposure, and outcomes. The AHRQ scale was scored based on 11 items, such as whether the data source was clear, whether the exposed and unexposed groups were listed, and whether the period for identifying patients was given. The maximum scores for NOS and AHRQ were 9 and 11, respectively. For NOS, a ≤6, 7–8, and 9 scores represent low, medium, and high quality, respectively. For AHRQ, a ≤3, 4–7, and 8–11 score represent low, medium, and high quality, respectively.

### 2.4. Statistical Analysis

“Review Manager” [RevMan, version 5.4 (The Cochrane Collaboration)] was used for the meta-analysis. Firstly, we pooled the means and standard deviations (SDs) of serum and RBC folate concentrations in the GDM and non-GDM groups. For studies that did not report the mean and SD values, the corresponding values were calculated from the median and interquartile interval [29]. Secondly, we extracted the multivariable-adjusted odds ratios (ORs) of the risk of GDM by taking the concentration of serum folate and RBC folate as continuous variables. We pooled the data using the generic inverse function of the “Review Manager” [RevMan, version 5.4 (The Cochrane Collaboration)]. Descriptive analyses were performed for data that could not be collected due to inconsistent reports. For the meta-analysis, considering that GDM women and non-GDM women are from different populations, a random effects model was presented for analysis. Sensitivity analysis was performed by excluding each study one by one to evaluate the credibility of the pooled results. A funnel plot was used to evaluate publication bias. We further conducted subgroup analysis on the results of serum folate concentration and RBC folate concentration according to the time of folate measurement to explore the source of heterogeneity or to evaluate the impact of grouping factors on the results.

## 3. Results

### 3.1. Study Characteristics

**Figure 1:**
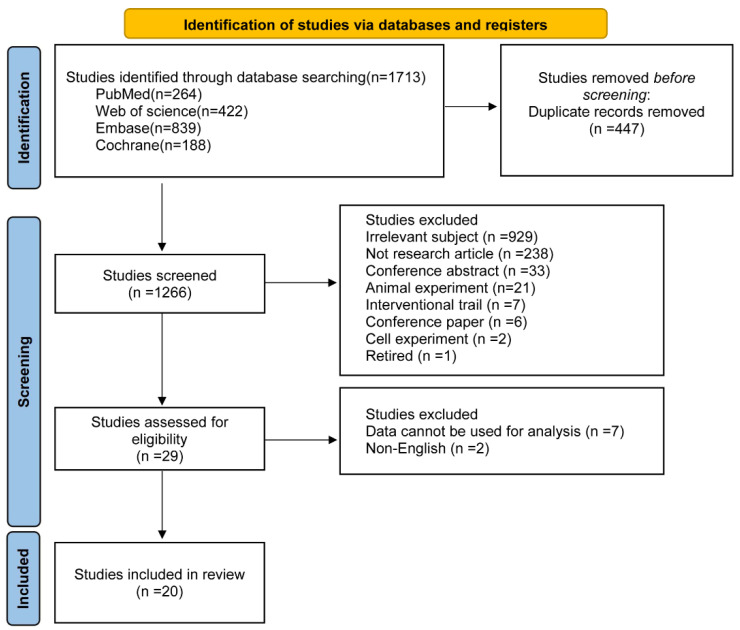
Flow diagram of the study selection process.

As shown in Figure 1 provides the research flow chart. In total, 1713 documents were retrieved from Pubmed, Embase, The Cochrane Library, and Web of Science databases, from which 29 studies were selected by removing duplicates and filtering titles and abstracts. After reviewing the full-text articles, we selected 20 [16,30,31,32,33,34,35,36,37,38,39,40,41,42,43,44,45,46,47,48], including 12 cohort studies, five cross-sectional studies, and three case-control studies. Among the selected studies, eight of the 20 were conducted in China, three in the United Kingdom, two in Turkey, and one in Canada, Singapore, India, Spain, Australia, Italy, and Poland, respectively. The sample sizes of the GDM ranged from 59 to 42,478.

The characteristics of the 20 studies are shown in Table 1. The data collection includes first author, year, country, study design, age, number of GDM, GDM criteria and quality score. Additionally, 5 data sets for RBC folate levels (Table 2), 17 data sets for serum folate levels (Table 2), and 12 data sets for multivariable-adjusted odds ratios (ORs) for serum/RBC folate levels and GDM risk (Table 3) are shown in Table 2 and Table 3.

### 3.2. Comparison of Serum and RBC Folate Levels between GDM and Non-GDM Women

**Figure 2:**
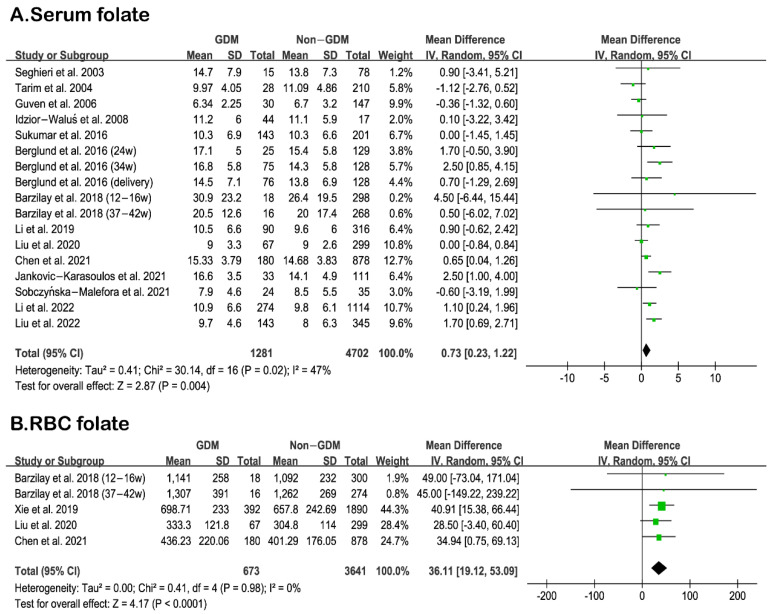
(**A**): Comparison of serum folate levels between GDM and Non-GDM. [33,34,35,37,38,39,41,42,43,44,45,46,47,48] (**B**): Comparison of RBC folate levels between GDM and Non-GDM [34,35,36,39]. Green squares represent MDs; horizontal lines indicate 95% confidence intervals; the black square indicates summary MDs with 95% confidence interval.

To explore the association between maternal folate status and GDM, the differences in serum/RBC folate levels were analyzed between GDM and Non-GDM groups. Fifteen studies were included for this analysis, among which the data of five RBC folate levels and seventeen serum/plasma folate levels could be extracted. For comparison, the folate concentration in all units was converted to ng/mL. Our analysis revealed that the serum folate concentration of GDM women was significantly higher than that of non-GDM women (MD: 0.73, 95% CI 0.23, 1.22, I^2^ = 47%, *p* = 0.004) (Figure 2). As shown in Figure 2, RBC folate concentration in GDM women was also higher than that in non-GDM women (MD: 36.11, 95% CI 19.12, 53.09, I^2^ = 0%, *p* < 0.0001). Sensitivity analyses were performed by excluding each study one by one, and all showed stable results. In addition, the funnel plot showed no publication bias. The funnel plot is shown in Figure S2 in the Supplementary Material.

**Figure 3:**
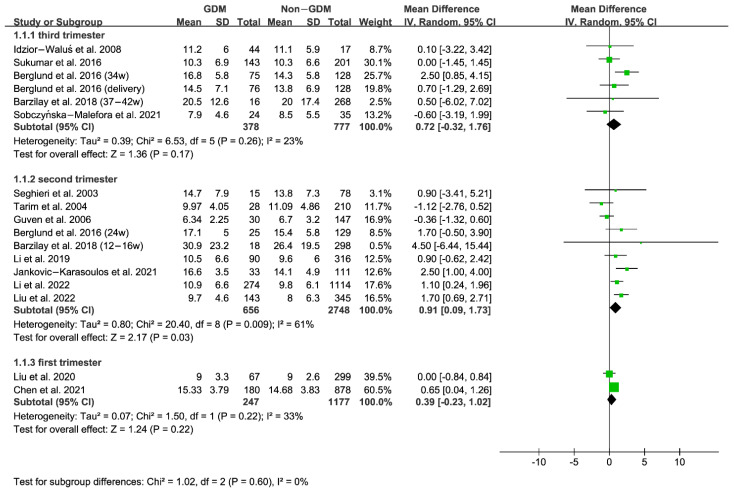
Serum folate: Subgroup analysis according to time of folate measurement [33,34,35,37,38,39,41,42,43,44,45,46,47,48]. Green squares represent MDs; horizontal lines indicate 95% confidence intervals; the black square indicates summary MDs with 95% confidence interval.

**Figure 4:**
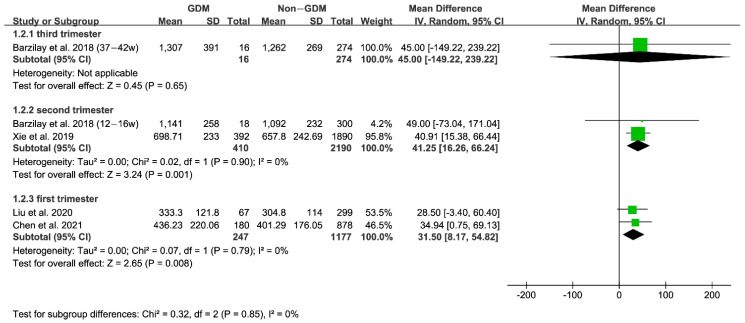
RBC folate: Subgroup analysis according to time of folate measurement [34,35,36,39]. Green squares represent MDs; horizontal lines indicate 95% confidence intervals; the black square indicates summary MDs with 95% confidence interval.

Furthermore, we performed subgroup analysis according to the time of folate measurement, as shown in Figure 3 and Figure 4. The subgroup results indicated that the serum folate concentration of GDM women in the second trimester of pregnancy was significantly higher than that of non-GDM women. In contrast, the RBC folate concentration in the first and second trimesters of pregnancy was remarkably higher than that of non-GDM women, suggesting the importance of monitoring serum or RBC folate levels during the first and second trimesters of pregnancy.

### 3.3. Relationship between Serum/RBC Folate and GDM Risk

**Figure 5:**
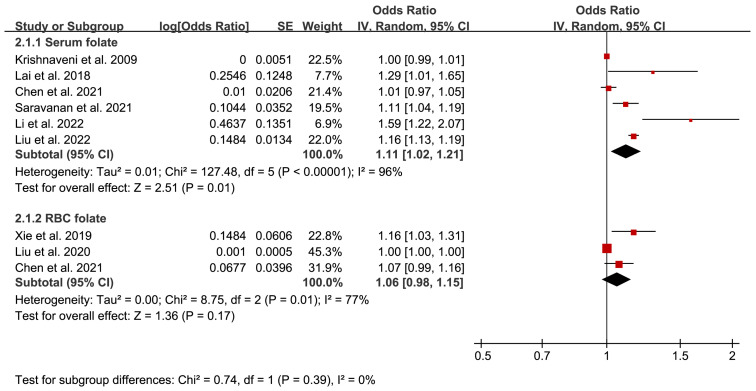
Adjusted odds ratios assessing the relationship between GDM and plasma/serum folate and RBC folate (as a continuous variable) [16,30,32,34,35,36,40,48]. Red squares represent ORs; horizontal lines indicate 95% confidence intervals; the black square indicates summary OR with 95% confidence interval.

To investigate the influence of serum/RBC folate levels on GDM risk, the data of ORs were extracted and analyzed from selected studies. As shown in Table 2, twelve 20 studies reported the multivariable-adjusted ORs of GDM risk. For serum and RBC folate, six and three adjusted ORs of GDM risk were continuous variables, respectively. We further conducted a combined analysis of adjusted ORs, showing that there was statistical significance between serum folate level and GDM risk (OR 1.11, 95% CI 1.02, 1.21, I^2^ = 96%, *p* = 0.01) (Figure 5), and no significant relationship between RBC folate level and GDM risk (OR 1.06, 95% CI 0.98, 1.15, I^2^ = 77%, *p* = 0.17) (Figure 5). Additionally, the sensitivity analysis was carried out by excluding studies one by one. In the study of the relationship between serum folate levels and GDM risk, the results were not statistically significant only after excluding the study of Li et al. [48]. Thus, the results were relatively stable, and the sensitivity analyses did not change their heterogeneity. However, since its heterogeneity was too high, we conducted a subgroup analysis based on the sample size, with a cut-off of 4000. The subgroup analysis forest plot can be seen in Figure S1 in the Supplementary Material. We found that the heterogeneity of the subgroup with a large sample size was 0%, and its results were statistically significant, while the heterogeneity of the subgroup with a small sample size was still large, with its results insignificant. This is because a larger sample size reflects the overall characteristics, and a smaller sample size results in insufficient representativeness of the population, making it difficult to ensure the accuracy and reliability of the results. The sample size may be the source of heterogeneity.

Similarly, the exclusion method was used to analyze the sensitivity analysis for the relationship between RBC folate levels and GDM risk. Notably, the heterogeneity was eliminated from 77% to 20% after excluding the study of Liu et al. [35]. Meanwhile, the result was also changed, showing that increased RBC folate level elevated GDM risk (OR 1.10, 95% CI 1.03, 1.17, I^2^ = 20%, *p* = 0.006). Through the analysis, we observed that the sample size of Liu et al.’s study was the smallest (*n* < 1000), whereas the sample size of the other two studies was more than 1000. Moreover, Liu et al.’s study had more correction factors for the OR, which may explain the inconsistent results on RBC folate levels and GDM risk among studies.

### 3.4. Descriptive Analysis

When defining the relationship between research variables and GDM risk, there were great differences in the classification methods of folate concentrations, which indicated that some studies could not be meta-analyzed. Therefore, we performed descriptive analyses for these studies.

For the serum folate levels, five [16,31,34,42,45] of ten studies reported no statistically significant correlation between the serum folate levels and the risk of GDM. The rest five studies demonstrated that a corresponding high serum folate level increased the risk of GDM. Li et al. [48] reported that the risk of GDM increased 2.28-fold (95% CI 1.49, 3.61) when the serum folate concentration was higher than 14.6 ng/mL. Besides, Liu et al. [30] found that the risk of GDM increased 1.54-fold (95% CI 1.40, 1.69) at high serum folate levels of 24.85 (24.05, 25.25) ng/mL. Likewise, Saravanan et al. [32] and Lai et al. [40] observed that with the increase of serum folate concentration, the risk of GDM increased 1.11-fold (95% CI 1.03, 1.18) and 1.29-fold (95% CI 1.01, 1.60), respectively. In line with this, Li et al. [47] showed the risk of GDM increased 1.98-fold (95% CI 1.00, 3.90) when the serum folate concentration was ≥12.2 ng/mL. Taken together, these data suggest that higher serum folate levels increase the risk of GDM.

For RBC folate levels, all three studies [34,35,36] reported that high RBC folate concentration significantly increased the risk of GDM. Chen et al. [34] found that when folate concentration is higher than 600 ng/mL, GDM risk increased 1.58-fold (95% CI 1.03, 2.41). Besides, Liu et al. [35] and Xie et al. [36] observed a risk of 2.47-fold (95% CI 1.01, 6.03) and 2.76-fold (95% CI 1.56, 4.89) with corresponding folate concentrations ≥380.7 ng/mL and ≥570.3 ng/mL, respectively, pointing toward higher RBC folate levels indeed increases the risk of GDM.

## 4. Discussion

Folic acid is widely used in clinic to prevent neural tube defects in fetuses. However, concerns have been raised recently about the potential adverse risks of high folate levels for mothers and children [4]. In this meta-analysis, our results demonstrated that serum and RBC folate levels in GDM mothers are higher than in non-GDM women. Additionally, combined with the result of the qualitative analysis, we can conclude that with the increase of serum and RBC folate concentration, the risk of GDM arises correspondingly. Moreover, subgroup analysis results showed that compared with the non-GDM women, women with GDM have higher serum folate levels in the second trimester (13–28 w) and elevated RBC folate levels in the first (1–12 w) and second trimesters (13–28 w). These results indicated that high serum and RBC folate levels increase the risk of pregnant women suffering from GDM. Additionally, considering GDM in most articles was found after 24 w, our results also suggest that higher RBC folate levels in mothers are found before GDM develops during their pregnancy. Hence it is important to evaluate and calculate the optimal RBC and plasma/serum folate levels at different stages of gestation to reduce the risk of GDM.

Concerning the mechanism of high folate status affecting GDM, the following possibilities have been proposed. Firstly, studies have indicated that unmetabolized folate in the blood may involve in the process of insulin resistance [18] or GDM [14,15] by reducing the toxicity of natural killer cells and leading to inflammation due to the imbalance of immune function [13]. Secondly, many studies have reported that folate and vitamin B12 may participate in the GDM process together [22,24,49]. As vitamin B12 and folate are crucial for synthesising protein and DNA, vitamin B12 deficiency alone or high folate that can exacerbate the effects of vitamin B12 deficiency can block DNA synthesis by inhibiting tetrahydrofolate production. Further, impaired mitochondrial DNA synthesis caused by vitamin B12 deficiency or high folate may lead to insulin resistance by triggering impaired insulin signaling through mitochondrial stress [16,17], thus leading to GDM. Indeed, vitamin B12 deficiency is associated with the development of GDM [49,50,51]. Lastly, studies have shown that elevated homocysteine levels are detrimental to pancreatic β cell metabolism and insulin secretion, likely triggering insulin resistance [52].

Interestingly, Maher et al. have found that high folate and low vitamin B12 may increase the risk of GDM through elevated homocysteine levels due to impaired methylation reactions and altered mitochondrial metabolism by methyl-trap [53]. Likewise, Selhub et al. have also demonstrated that in vitamin B12 deficiency, high folate is associated with increased total homocysteine in clinical studies [54]. Inline, Cho et al. [55] have found that compared with the Non-GDM group, women with GDM have elevated levels of homocysteine, hereby suggesting that high folate may trigger the development of GDM via elevated homocysteine level under the deficiency of vitamin B12. Altogether, it is likely that high folate concentrations are involved in the development of GDM not only by imbalanced immune function but also by exacerbating vitamin B12 deficiency and elevated homocysteine.

In addition, genetic factors are also risk elements for GDM. It has been found that the MTHFR gene, a key regulatory enzyme of folate metabolism [56], can affect the relationship between folate and GDM risk [35]. Liu et al. found for the first time that rs1801133 (MTHFR C677T) polymorphism may affect the association between RBC folate and GDM risk by affecting the folate status [35]. Besides, Li et al. analyzed the association between the MTHFR rs1801131 genotype and GDM in Chinese pregnant women by gene stratification. Results demonstrated that the association between folate and GDM was more obvious in pregnant women with the MTHFR rs1801131 TT genotype than in pregnant women with the MTHFR rs1801131 G allele [48]. Therefore, it is necessary to conduct extensive research to determine the impact of MTHFR gene polymorphism on folate metabolism and GDM risk in the future. And taking appropriate folic acid supplementation for pregnant women with the corresponding genotype may bring new ideas for GDM prevention.

In line with our findings, meta-analyses have shown that high maternal folate is associated with a higher risk of GDM [23,24,25]. However, unlike our subgroup analysis result, Yang et al. demonstrated that women with GDM had higher folate levels in the second or third trimester [23]. The discrepancy may be explained by the fact that Yang et al. did not include the measurement data of folate in early pregnancy. Additionally, some limitations existed in our study, leading us to make conclusions cautiously. For instance, there are certain differences in the diagnostic time and diagnostic criteria for GDM and methods to measure folate concentration, which may affect the results. Besides, when comparing serum and RBC folate levels between GDM and non-GDM women, most folate levels were measured in the middle and late pregnancy, with only a few corresponding reports in early pregnancy.

Moreover, several studies were included in the article with a NOS score of 6, representing the possible low quality of their studies. However, the NOS scale is scored based on three aspects of study object selection, including comparability, exposure, and outcomes, which varies widely and subjectively. The low NOS scale in our study might generate from the strictly standardized research process or individuals. Considering other relevant meta-analysis studies have included these three articles, the quality of these articles is appropriate for meta-analysis. Hence, we also included these three studies with a NOS score 6 in our meta-analysis.

Additionally, there are several inconsistencies among studies regarding the adjusted factors in the multivariable analysis, which may influence the results. Finally, although there was a statistically significant difference in folate levels between GDM and non-GDM, this difference was relatively small, and the adjusted RBC folate OR did not differ between GDM and non-GDM women, which also weakens the hypothesis that high levels of folate in the first trimester are associated with GDM. Coupled with the fact that the higher dose of folic acid (5 mg/day), the better reduction in fetal malformations (from 15% to 85%) [57], folate decreased fetal malformations other than NTD, such as congenital heart defects, obstructive urinary tract anomalies, limb deficiencies, orofacial clefts, congenital hypertrophic pyloric stenosis [58] and trisomy 21 [59], future studies should focus on balancing between the risk of GDM and the favourable effects of folic acid.

Although there are some limitations existed, our study also has several advantages. For instance, we have extracted more valuable data on serum folate levels and RBC folate levels for determining the effect of folate on the development of GDM rather than the intake levels of folic acid supplements. Then, compared with other meta-analysis studies with similar topics, we included 12 newly published and non-included articles, which made our analysis more complete and updated. Furthermore, our findings further demonstrated for the first time that women with GDM have higher serum folate levels in the second trimester and elevated RBC folate levels in the first and second trimesters than the non-GDM group, which is also a novel finding compared with other meta-analyses.

## 5. Conclusions

In conclusion, our results show that the risk of GDM is related to high serum/plasma and RBC folate levels, suggesting that high maternal serum or RBC folate status may indicate a higher risk of GDM. Moreover, according to our subgroup analysis, we have observed that serum/plasma folate levels in the second trimester of pregnancy and RBC folate levels in the first and second trimesters of pregnancy in the GDM group were significantly higher than those of the non-GDM group. Future studies should determine the recommended folic acid cutoff balancing the risk for GDM and fetal malformations. As folic acid supplementation is widely used clinically, our findings provide a new perspective for clinicians to rebalance the effects of folate on pregnant women. However, more studies are needed to clarify the possible mechanisms by which high folate concentrations increase the risk of GDM.

## Figures and Tables

**Table 1:** Characteristics of included studies.

ID	First Author	Country	Study Design	Sample Size	GDM(n)	Age	Test for GDM	GDM Criteria	Period for GDM Assessment	Quality Score
1	Seghieri et al., 2003 [43]	Italy	Cross-sectional	93	15	GDM: 34.6 ± 3.1, Control: 32.3 ± 3.7	100 g OGTT	the American Diabetes Association	24–28 weeks	6
2	Tarim et al., 2004 [37]	Turkey	Prospective cohort	238	28	GDM: 32 ± 4.03, Control: 26.83 ± 4.44	50 g OGTT	Carpenter and Coustan	24–28 weeks	6
3	Guven et al., 2006 [38]	Turkey	Cross-sectional	177	30	GDM: 30.0 ± 4.3, Control: 28.6 ± 3.4	100 g OGTT	Carpenter and Coustan	24–28 weeks	7
4	Idzior-Waluś et al., 2008 [44]	Poland	Prospective cohort	61	44	GDM: 30.5 ± 6.6, Control: 26.2 ± 4.0	75 g OGTT	WHO1999	26–32 weeks	8
5	Krishnaveni et al., 2009 [16]	India	Prospective cohort	785	49	23 ± 4.5	100 g OGTT	Carpenter-Coustan criteria	32 ± 2 weeks	6
6	Sukumar et al., 2016 [45]	UK	Case-control	344	143	GDM: 31.4 ± 5.8, Control: 29.6 ± 5.9	75 g OGTT	WHO1999	24–36 weeks	7
7	Berglund et al., 2016 [41]	Spain	Prospective cohort	331	76	GDM: 33.7 ± 4.6	NA	NDDG	24 weeks, 34 weeks, delivery	7
8	Barzilay et al., 2018 [39]	Canada	Prospective cohort	368	16	GDM: 34.4 ± 5.3, Control: 32.1 ± 4.8	50 g OGTT	Canadian Diabetes Association 2008 practice guidelines	24–28 weeks	6
9	Lai et al., 2018 [40]	Singapore	Cross-sectional	913	164	<35, *n* = 705, ≥35, *n* = 208	75 g OGTT	1999 World Health Organization standard criteria	26–28 weeks	8
10	Xie et al., 2019 [36]	China	Prospective cohort	2282	392	GDM: 29.01 ± 3.15, Control: 27.89 ± 3.18	75 g OGTT	FPG ≥ 5.5 mmol/L, 2-h plasma glucose ≥ 8 mmol/L	24–28 weeks	9
11	Li et al., 2019 [47]	China	Cross-sectional	406	90	29.4 ± 4.5	75 g OGTT	IADPSG	24–28 weeks	8
12	Liu et al., 2020 [35]	China	Prospective cohort	366	67	GDM: 30.5 ± 4.0, Control: 28.9 ± 3.5	75 g OGTT	IADPSG	24–28 weeks	8
13	Jankovic-Karasoulos et al., 2021 [42]	Australia	Prospective cohort	144	33	GDM: 28.9 ± 5.2, Control: 27.9 ± 5.9	NA	WHO 2016	Around 26 weeks	7
14	Saravanan et al., 2021 [32]	UK	Prospective cohort	4746	NICE-GDM: 538, IADPSG-GDM: 633	30.51 ± 5.29	75 g OGTT	NICE, IADPSG	26–28 weeks	9
15	Sobczyńska-Malefora et al., 2021 [33]	UK	Cross-sectional	59	24	GDM: 30.8 ± 5.2, Control: 27.7 ± 4.8	75 g OGTT	Local diagnostic	28 weeks	7
16	Chen et al., 2021 [34]	China	Prospective cohort	1058	180	30.24 ± 3.97	75 g OGTT	IADPSG	24–28 weeks	8
17	Liu et al., 2022 [30]	China	Retrospective cohort	42,478	5122	NA	75 g OGTT	IADPSG	24–28 weeks	7
18	Yuan et al., 2022 [31]	China	Retrospective cohort	11,549	965	NA	NA	NA	NA	8
19	Liu et al., 2022 [46]	China	Case-control	488	143	GDM: 30.63 ± 4.64, Control: 28.51 ± 4.44	75 g OGTT	IADPSG	24–28 weeks	8
20	Li et al., 2022 [48]	China	Case-control	1388	274	<30, *n* = 692, 30–35, *n* = 489, ≥35, *n* = 207	75 g OGTT	IADPSG	24–28 weeks	7

Abbreviations: GDM, gestational diabetes mellitus; NA, not available; IADPSG, International Association of the Diabetes and Pregnancy Study Groups; OGTT, oral glucose tolerance test; NICE, National Institute for Health and Clinical Excellence; WHO, World Health Organization; NDDG, the National Diabetes Data Group; FPG, fasting plasma glucose; h, hour.

**Table 2:** Comparison between GDM and non-GDM according to folate level.

ID	First Author	Folate Level (ng/mL)		Folate Status	Time for Folate Measurement
		GDM	Non-GDM		
1	Seghieri et al., 2003 [43]	14.7 ± 7.9	13.8 ± 7.3	serum folate	24–28 weeks gestation (second trimester)
2	Tarim et al., 2004 [37]	9.97 ± 4.05	11.09 ± 4.86	serum folate	24–28 weeks gestation (second trimester)
3	Guven et al., 2006 [38]	6.34 ± 2.25	6.7 ± 3.2	serum folate	24–28 weeks gestation (second trimester)
4	Idzior-Waluś et al., 2008 [44]	11.2 ± 6	11.1 ± 5.9	serum folate	26–32 weeks gestation (third trimester)
5	Berglund et al., 2016 [41]	17.1 ± 5.0	15.4 ± 5.8	serum folate	24 weeks gestation (second trimester)
		16.8 ± 5.8	14.3 ± 5.8	serum folate	34 weeks gestation (third trimester)
		14.5 ± 7.1	13.8 ± 6.9	serum folate	delivery (third trimester)
6	Sukumar et al., 2016 [45]	10.3 ± 6.9	10.3 ± 6.6	serum folate	24–36 weeks gestation (third trimester)
7	Barzilay et al., 2018 [39]	30.9 ± 23.2	26.4 ± 19.5	serum folate	12–16 weeks gestation (second trimester)
		20.5 ± 12.6	20 ± 17.4	serum folate	37–42 weeks gestation (third trimester)
		1141 ± 258	1092 ± 232	RBC folate	12–16 weeks gestation (second trimester)
		1307 ± 391	1262 ± 269	RBC folate	37–42 weeks gestation (third trimester)
8	Xie et al., 2019 [36]	698.71 ± 233	657.80 ± 242.69	RBC folate	19–24 weeks gestation (second trimester)
9	Li et al., 2019 [47]	10.5 ± 6.6	9.6 ± 6.0	serum folate	24–28 weeks gestation (second trimester)
10	Liu et al., 2020 [35]	9.0 ± 3.3	9.0 ± 2.6	serum folate	before 12 weeks gestation (first trimester)
		333.3 ± 121.8	304.8 ± 114	RBC folate	before 12 weeks gestation (first trimester)
11	Sobczyńska-Malefora et al., 2021 [33]	7.9 ± 4.6	8.5 ± 5.5	serum folate	28 weeks gestation (third trimester)
12	Chen et al., 2021 [34]	436.23 ± 220.06	401.29 ± 176.05	RBC folate	9–13 weeks gestation (first trimester)
		15.33 ± 3.79	14.68 ± 3.83	serum folate	9–13 weeks gestation (first trimester)
13	Jankovic-Karasoulos et al., 2021 [42]	16.6 ± 3.5	14.1 ± 4.9	serum folate	15 ± 1 weeks gestation (second trimester)
14	Liu et al., 2022 [46]	9.7 ± 4.6	8.0 ± 6.3	serum folate	24–28 weeks gestation (second trimester)
15	Li et al., 2022 [48]	10.9 ± 6.6	9.8 ± 6.1	serum folate	24–28 weeks gestation (second trimester)

Data are presented as mean ± SD—abbreviations: GDM, gestational diabetes mellitus.

**Table 3:** Association between folate status (RBC/serum) and GDM risk.

ID	First Author	Outcome	Adjusted OR (95% CI)	Adjusted Factors	Time for Measurement
1	Krishnaveni et al., 2009 [16]	Serum folate As continuous variable	1.0 (0.99, 1.0)	age, religion, socioeconomic status, parity and family history of diabetes	30 ± 2 weeks gestation
2	Sukumar et al., 2016 [45]	Serum folate: ng/mL 3.1–18.7 <3.1	Reference 0.89 (0.07, 11.38)	age, parity, ethnic origin, smoking, the gestation of bloods, serum B12, gestational BMI	24–36 weeks gestation
3	Lai et al., 2018 [40]	Serum folate As continuous variable	1.29 (1.01, 1.60)	maternal age, ethnicity, education, income, smoking, alcohol intake, physical activity, pre-pregnancy BMI, parity, family history of diabetes, and previous occurrence of GDM, plasma B6 and B12	at 26 weeks gestation
4	Xie et al., 2019 [36]	RBC folate: ng/mL Q1: <398.6 Q2: 398.6–570.3 Q3: ≥570.3 As continuous variable	Reference 2.17 (1.20, 3.95) 2.76 (1.56, 4.89) 1.16 (1.03, 1.30)	maternal age, parity, and BMI	second trimester
5	Li et al., 2019 [47]	Serum folate: ng/mL Q1: <6.9 Q2: 6.9–12.2 Q3: ≥12.2	Reference 1.12 (0.59, 2.13) 1.98 (1.00, 3.90)	age, ethnicity, education, parity, pp-BMI, family history of diabetes, serum vitamin B12 concentrations	24–28 weeks gestation
6	Liu et al., 2020 [35]	RBC folate: ng/mL Q1: <224.7 Q2: 224.7–286.0 Q3: 286.0–380.7 Q4: ≥380.7 As continuous variable	Reference 1.35 (0.53, 3.45) 1.37 (0.54, 3.45) 2.47 (1.01, 6.03) 1.001(1.000, 1.002)	age, physical activity, BMI, parity, family history of diabetes, use of folic acid supplements, HOMA-IR, C-reactive protein, hemoglobin, vitamin B12, and serum homocysteine	early pregnancy
7	Saravanan et al., 2021 [32]	Serum folate As continuous variable	1.11 (1.03, 1.18)	age, parity, smoking status, ethnicity, family history, household income and B12 status	early pregnancy
8	Chen et al., 2021 [34]	Serum folate: ng/mL Q1: <13.9 Q2: 13.9–16.0 Q3: ≥16.0 As continuous variable	Reference 0.91 (0.58, 1.44) 1.36 (0.94, 1.99) 1.01 (0.97, 1.05)	age, pre-conceptional BMI, family history of diabetes, smoking exposure, and drinking status.	Early pregnancy (9–13 weeks)
		RBC folate: ng/mL Q1: <400 Q2: 400–600 Q3: ≥600 As continuous variable	Reference 1.39 (0.94, 2.04) 1.58 (1.03, 2.41) 1.07 (0.99, 1.15)		
9	Jankovic-Karasoulos et al., 2021 [42]	Serum folate effect for every 5-unit increase	1.22 (0.93, 1.59)	maternal age, BMI, smoking status	15 ± 1 weeks gestation
10	Liu et al., 2022 [30]	Serum folate: ng/mL Q1: 11.07 (8.82, 12.81) Q2: 17.14 (15.75, 18.44) Q3: 22.23 (20.71, 23.24) Q4: 24.85 (24.05, 25.25) As continuous variable	Reference 1.15 (1.04, 1.26) 1.40 (1.27, 1.54) 1.54 (1.40, 1.69) 1.16 (1.13, 1.19)	pre-pregnancy BMI status, fetal gender, parity, maternal age, vitamin B12 level and maternal education	before 24 weeks gestation
11	Yuan et al., 2022 [31]	Serum folate P5–P95 >P95 <P5	Reference 1.23 (0.99, 1.53) 0.40 (0.23, 0.70)	maternal age, BMI, gravidity, parity, SVB12 levels	at delivery
12	Li et al., 2022 [48]	Serum folate: ng/mL Q1: <6.2 Q2: 6.2–9.4 Q3: 9.4–14.6 Q4: ≥14.6 As continuous variable	Reference 1.47 (0.99, 2.26) 1.61 (1.07, 2.49) 2.28 (1.49, 3.61) 1.59 (1.22, 2.13)	age, ethnicity, education, drinking, smoking, parity, family history of diabetes, pre-pregnancy BMI, serum B12 and Hcy concentrations	24–28 weeks gestation

Abbreviations: BMI, body mass index; OR, odds ratio; CI, confidence interval; Q, quartile; HOMA-IR, homeostasis model assessment-insulin resistance; Hcy, homocysteine; SVB12, serum vitamin B12; pp-BMI, pre-pregnancy body mass index.

## Data Availability

No new data were created in this meta-analysis.

## Supplementary Materials

The following supporting information can be downloaded at: https://www.mdpi.com/article/10.3390/nu15122766/s1.
