# Supplementary material for: The Influence of Maternal Folate Status on Gestational Diabetes Mellitus: A Systematic Review and Meta-Analysis
Source: Nutrients. 2023 Jun 16;15(12):2766. doi: 10.3390/nu15122766 (PMC10300922; doi:10.3390/nu15122766)
Supplement: Supplementary file 1 [file nutrients-15-02766-s001.zip › Supplemental tables.pdf]

Supplemental tables

Table S1: Search terms.

	Subject headings	Free words
Pubmed	diabetes,gestational	Diabetes, Pregnancy-Induced<br>Diabetes, Pregnancy Induced<br>Pregnancy-Induced Diabetes<br>Gestational Diabetes<br>Diabetes Mellitus, Gestational<br>Gestational Diabetes Mellitus
	folic acid	Vitamin M<br>Vitamin B9<br>B9, Vitamin<br>Pteroylglutamic Acid<br>Folic Acid, Monopotassium Salt<br>Folic Acid, Monosodium Salt<br>Folic Acid, Potassium Salt<br>Folic Acid, (DL)-Isomer<br>Folvite<br>Folacin<br>Folate<br>Folic Acid, (D)-Isomer<br>Folic Acid, Calcium Salt (1:1)<br>Folic Acid, Sodium Salt
Embase	Pregnancy diabetes mellitus	diabetes mellitus gravidarum;<br>diabetes, gestational;<br>diabetes, pregnancy;<br>gestational diabetes;<br>gestational diabetes mellitus;<br>pregnancy diabetes;<br>pregnancy in diabetics
	Folic acid	2 [[4 [(2 amino 4 oxo 3h pteridin 6 yl) methylamino] benzoyl] amino] pentanedioic acid;<br>2 amino 6 [[4 [(1, 3 dicarboxypropyl) aminocarbonyl] phenylamino] methyl] 4 pteridinol;<br>2 amino 6 [[4 [(1, 3 dicarboxypropyl) carbamoyl] anilino] methyl] 4 pteridinol;<br>acfol;<br>acide folique ccd;<br>acido folico;<br>acifolic;<br>apo-folic;<br>filicine;

		folacid;<br>folacin;<br>folart;<br>folate;<br>folate acid;<br>folate sodium;<br>folavit;<br>foldine;<br>foliamin;<br>folic acid dha;<br>folicet;<br>folicid;<br>folina;<br>folinsyre;<br>folitab;<br>folium acid;<br>folivit;<br>folsan;<br>folsav;<br>folverlan;<br>folvite;<br>gravi-fol;<br>ingafol;<br>lactobacillus casei factor;<br>lafol;<br>lexpec;<br>megafol;<br>mission prenatal;<br>n [4 [[(2 amino 3, 4 dihydro 4 oxo 6 pteridinyl) methyl] amino] benzoyl] glutamic acid;<br>n [[4 [[(2 amino 4 oxo 1, 4 dihydro 6 pteridinyl) methyl] amino] phenyl] carbonyl] glutamic acid;<br>n [[4 [[(2 amino 4 oxo 1, 4 dihydropteridin 6 yl) methyl] amino] phenyl] carbonyl] glutamic acid;<br>n [para [(2 amino 4 hydroxy 6 pteridylmethyl) amino] benzoyl] glutamic acid;<br>neocepri;<br>nsc 3073;<br>pteroyglutamic acid tablets;<br>pteroyl glutamate;<br>pteroyl l glutamic acid;

		pteroyl monoglutamate;<br>pteroylglutamate;<br>pteroylglutamic acid;<br>pteroylmonoglutamate;<br>pteroylmonoglutamic acid;<br>rubiefol;<br>sodium folate;<br>speciafoldine;<br>unifol (folic acid);<br>vifolin;<br>vitamin bc;<br>vitamin m
Cochrane	Diabetes, Gestational	Diabetes, Pregnancy-Induced;<br>Gestational Diabetes Mellitus;<br>Gestational Diabetes;<br>Diabetes Mellitus, Gestational;<br>Pregnancy-Induced Diabetes;<br>Diabetes, Pregnancy Induced
	Folic Acid	Folic Acid, Potassium Salt;<br>Folate;<br>Folvite;<br>Folacin;<br>Folic Acid, Monosodium Salt;<br>Folic Acid, Calcium Salt (1:1);<br>Folic Acid, Sodium Salt;<br>Folic Acid, Monopotassium Salt;<br>Pteroylglutamic Acid;<br>Vitamin B9;<br>Vitamin M;<br>B9, Vitamin;<br>Folic Acid, (D)-Isomer;<br>Folic Acid, (DL)-Isomer

Table S2: Pubmed search strategy.

(("Diabetes, Gestational"[Mesh]) OR (((((Diabetes, Pregnancy-Induced) OR (Diabetes, Pregnancy Induced)) OR (Pregnancy-Induced Diabetes)) OR (Gestational Diabetes)) OR (Diabetes Mellitus, Gestational)) OR (Gestational Diabetes Mellitus))) AND (("Folic Acid"[Mesh]) OR (((((((((((((Vitamin M) OR (Vitamin B9)) OR (B9, Vitamin)) OR (Pteroylglutamic Acid)) OR (Folic Acid, Monopotassium Salt)) OR (Folic Acid, Monosodium Salt)) OR (Folic Acid, Potassium Salt)) OR (Folic Acid, (DL)-Isomer)) OR (Folvite)) OR (Folacin)) OR (Folate)) OR (Folic Acid, (D)-Isomer)) OR (Folic Acid, Calcium Salt (1:1))) OR (Folic Acid, Sodium Salt)))

Table S3: Web of Science search strategy.

Web of Science Search Strategy (v0.1)<br># Databases: All databases<br># Permissions:<br>- WOS: 1985 to 2022<br>- CSCD: 2020 to 2022<br>- DIIDW: 1966 to 2022<br>- KJD: 1980 to 2022<br>- SCIELO: 2002 to 2022
Search.<br>1: ((((((TS=(diabetes,gestational)) OR TS=(Diabetes, Pregnancy-Induced)) OR TS=(Diabetes, Pregnancy Induced)) OR TS=(Pregnancy-Induced Diabetes)) OR TS=(Gestational Diabetes)) OR TS=(Diabetes Mellitus, Gestational)) OR TS=(Gestational Diabetes Mellitus) Date of operation: Tue Oct 18 2022 20:17:27 GMT+0800 (China Standard Time) Search results: 32950<br><br>2: (((((((((((((TS=(folic acid)) OR TS=(Vitamin M)) OR TS=(Vitamin B9)) OR TS=(B9, Vitamin)) OR TS=(Pteroylglutamic Acid)) OR TS=(Folic Acid, Monopotassium Salt)) OR TS=(Folic Acid, Monosodium Salt)) OR TS=(Folic Acid, Potassium Salt)) OR TS=(Folic Acid, (DL)-Isomer)) OR TS=(Folvite)) OR TS=(Folacin)) OR TS=(Folate)) OR TS=(Folic Acid, (D)-Isomer)) OR TS=(Folic Acid, Calcium Salt (1:1))) OR TS=(Folic Acid, Sodium Salt) Date of operation: Tue Oct 18 2022 20:21:41 GMT+0800 (China Standard Time) Search results: 93182<br><br>3: #2 AND #1 Running date: Tue Oct 18 2022 20:22:12 GMT+0800 (CST) Search result: 422

Table S4: Cochrane search strategy.

Search Name:		
Date Run: 16/10/2022 08:25:43		
Comment:		
ID	Search	Hits
#1	MeSH descriptor: [Diabetes, Gestational] explode all trees	1165
#2	Diabetes, Pregnancy-Induced	254
#3	Gestational Diabetes Mellitus	3047
#4	Gestational Diabetes	4125
#5	Diabetes Mellitus, Gestational	3047
#6	Pregnancy-Induced Diabetes	254
#7	Diabetes, Pregnancy Induced	588
#8	#1 or #2 or #3 or #4 or #5 or #6 or #7	4400
#9	MeSH descriptor: [Folic Acid] explode all trees	3764
#10	Folic Acid, Potassium Salt	21
#11	Folate	2671
#12	Folvite	10
#13	Folacin	23
#14	Folic Acid, Monosodium Salt	4
#15	Folic Acid, Sodium Salt	35
#16	Folic Acid, Monopotassium Salt	1
#17	Pteroylglutamic Acid	18
#18	Vitamin B9	81
#19	Vitamin M	11976
#20	B9, Vitamin	81
#21	#9 or #10 or #11 or #12 or #13 or #14 or #15 or #16 or #17 or #18 or #19 or #20	16746
#22	#8 and #21	188

Table S5: Embase search strategy.

Session Results		
No. Query Results		Results Date
#70. #9 AND #69	839	19 Oct 2022
#69. #10 OR #11 OR #12 OR #13 OR #14 OR #15 OR #16 OR #17 OR #18 OR #19 OR #20 OR #21 OR #22 OR #23 OR #24 OR #25 OR #26 OR #27 OR #28 OR #29 OR #30 OR #31 OR #32 OR #33 OR #34 OR #35 OR #36 OR #37 OR #38 OR #39 OR #40 OR #41 OR #42 OR #43 OR #44 OR #45 OR #46 OR #47 OR #48 OR #49 OR #50 OR #51 OR #52 OR #53 OR #54 OR #55 OR #56 OR #57 OR #58 OR #59 OR #60 OR #61 OR #62 OR #63 OR #64 OR #65 OR #66 OR #67 OR #68	83,593	19 Oct 2022
#68. 'vitamin m':ab,ti	16	19 Oct 2022
#67. 'vitamin bc':ab,ti	22	19 Oct 2022
#66. 'vifolin':ab,ti	1	19 Oct 2022
#65. 'unifol (folic acid)':ab,ti		19 Oct 2022
#64. 'speciafoldine':ab,ti		19 Oct 2022
#63. 'sodium folate':ab,ti	11	19 Oct 2022
#62. 'rubiefol':ab,ti		19 Oct 2022
#61. 'pteroylmonoglutamic acid':ab,ti	48	19 Oct 2022
#60. 'pteroylmonoglutamate':ab,ti	55	19 Oct 2022
#59. 'pteroylglutamic acid':ab,ti	405	19 Oct 2022
#58. 'pteroylglutamate':ab,ti	54	19 Oct 2022
#57. 'pteroyl monoglutamate':ab,ti		19 Oct 2022
#56. 'pteroyl l glutamic acid':ab,ti	11	19 Oct 2022
#55. 'pteroyl glutamate':ab,ti	12	19 Oct 2022
#54. 'pteroyglutamic acid tablets':ab,ti		19 Oct 2022
#53. 'nsc 3073':ab,ti		19 Oct 2022
#52. 'neocepri':ab,ti		19 Oct 2022
#51. 'n [para [(2 amino 4 hydroxy 6 pteridylmethyl) amino] benzoyl] glutamic acid':ab,ti		19 Oct 2022

#50. 'n [[4 [[(2 amino 4 oxo 1, 4 dihydropteridin 6 yl) methyl] amino] phenyl] carbonyl] glutamic acid':ab,ti		19 Oct 2022
#49. 'n [[4 [[(2 amino 4 oxo 1, 4 dihydro 6 pteridinyl) methyl] amino] phenyl] carbonyl] glutamic acid':ab,ti		19 Oct 2022
#48. 'n [4 [[(2 amino 3, 4 dihydro 4 oxo 6 pteridinyl) methyl] amino] benzoyl] glutamic acidl':ab,ti		19 Oct 2022
#47. 'mission prenatal':ab,ti	1	19 Oct 2022
#46. 'megafol':ab,ti	5	19 Oct 2022
#45. 'lexpec':ab,ti		19 Oct 2022
#44. 'lafol':ab,ti	1	19 Oct 2022
#43. 'lactobacillus casei factor':ab,ti	18	19 Oct 2022
#42. 'ingafol':ab,ti		19 Oct 2022
#41. 'gravi-fol':ab,ti		19 Oct 2022
#40. 'folvite':ab,ti	6	19 Oct 2022
#39. 'folverlan':ab,ti		19 Oct 2022
#38. 'folsav':ab,ti	1	19 Oct 2022
#37. 'folsan':ab,ti	7	19 Oct 2022
#36. 'folivit':ab,ti		19 Oct 2022
#35. 'folium acid':ab,ti	1	19 Oct 2022
#34. 'folitab':ab,ti		19 Oct 2022
#33. 'folinsyre':ab,ti	2	19 Oct 2022
#32. 'folina':ab,ti	2	19 Oct 2022
#31. 'folicid':ab,ti		19 Oct 2022
#30. 'folicet':ab,ti		19 Oct 2022
#29. 'folic acid dha':ab,ti	1	19 Oct 2022
#28. 'foliamin':ab,ti	1	19 Oct 2022
#27. 'foldine':ab,ti		19 Oct 2022
#26. 'folavit':ab,ti		19 Oct 2022
#25. 'folate sodium':ab,ti	5	19 Oct 2022
#24. 'folate acid':ab,ti	71	19 Oct 2022
#23. 'folate':ab,ti	37,791	19 Oct 2022
#22. 'folart':ab,ti		19 Oct 2022
#21. 'folacin':ab,ti	269	19 Oct 2022
#20. 'folacid':ab,ti		19 Oct 2022
#19. 'filicine':ab,ti	1	19 Oct 2022

#18.	'apo-folic':ab,ti		19 Oct 2022
#17.	'acifolic':ab,ti		19 Oct 2022
#16.	'acido folico':ab,ti	52	19 Oct 2022
#15.	'acide folique ccd':ab,ti		19 Oct 2022
#14.	'acfol':ab,ti	7	19 Oct 2022
#13.	'2 amino 6 [[4 [(1, 3 dicarboxypropyl) carbamoyl] anilino] methyl] 4 pteridinol':ab,ti		19 Oct 2022
#12.	'2 amino 6 [[4 [(1, 3 dicarboxypropyl) aminocarbonyl] phenylamino] methyl] 4 pteridinol':ab,ti		19 Oct 2022
#11.	'2 [[4 [(2 amino 4 oxo 3h pteridin 6 yl) methylamino] benzoyl] amino] pentanedioic acid':ab,ti		19 Oct 2022
#10.	'folic acid'/exp	71,858	19 Oct 2022
#9.	#1 OR #2 OR #3 OR #4 OR #5 OR #6 OR #7 OR	49,213	19 Oct 2022
#8.	'pregnancy in diabetics':ab,ti	29	19 Oct 2022
#7.	'pregnancy diabetes':ab,ti	460	19 Oct 2022
#6.	'gestational diabetes mellitus':ab,ti	15,568	19 Oct 2022
#5.	'gestational diabetes':ab,ti	30,050	19 Oct 2022
#4.	'diabetes, pregnancy':ab,ti	391	19 Oct 2022
#3.	'diabetes, gestational':ab,ti	415	19 Oct 2022
#2.	'diabetes mellitus gravidarum':ab,ti	2	19 Oct 2022
#1.	'pregnancy diabetes mellitus'/exp	46,239	19 Oct 2022

Table S6: Newcastle-Ottawa Scale (NOS) for assessing the quality of included cohort studies (n=12).

Study	Selection				Comparability	Exposure			Score
	Representativeness of the exposed cohort	Selection of the non-exposed cohort	Ascertainment of exposure	Demonstration that outcome of interest was not present at the start of the study	Comparability of cohorts on the basis of the design or analysis	Assessment of outcome	Length of follow up	Adequacy of follow up of cohorts	
Liu et al. 2022 [30]	1	1	1	1	1	1	1	0	7
Yuan et al. 2022 [31]	1	1	1	1	2	0	1	1	8
Saravanan et al. 2021 [32]	1	1	1	1	2	1	1	1	9
Chen et al. 2021 [34]	1	0	1	1	2	1	1	1	8
Liu et al. 2020 [35]	1	1	1	1	1	1	1	1	8
Xie et al. 2019 [36]	1	1	1	1	2	1	1	1	9
Tarim et al. 2004 [37]	1	1	1	1	0	1	1	0	6
Barzilay et al. 2018 [39]	1	1	1	1	1	0	1	0	6

Krishnaveni et al. 2009 [16]	1	1	1	1	0	1	1	0	6
Berglund et al. 2016 [41]	1	1	0	1	1	1	1	1	7
Jankovic-Karasoulos et al. 2021 [42]	1	1	1	1	1	1	1	0	7
Idzior-Waluś et al. 2008 [44]	1	1	1	1	1	1	1	1	8

Table S7: Newcastle-Ottawa Scale (NOS) for assessing the quality of included case-control studies (n=3).

Study	Selection				Comparability	Exposure			score
	Adequacy of case definition	Representativeness of the cases	Selection of controls	Definition of controls	Comparability of cases and controls on the basis of the design or analysis	Ascertainment of exposure	Same method of ascertainment for cases and controls	Non-Response rate	
Li et al. 2022 [48]	1	1	1	0	1	1	1	1	7
Liu et al. 2022 [46]	1	1	1	1	1	1	1	1	8
Sukumar et al. 2016 [45]	1	1	1	1	1	1	0	1	7

Table S8: Agency for Healthcare Research and Quality (AHRQ) for assessing the quality of included cross-sectional studies (n=5).

Study	Define the source of information	List inclusion and exclusion criteria for exposed and unexposed subjects or refer to previous publications	Indicate time period used for identifying patients	Indicate whether or not subjects were consecutive if not population-based	Indicate if evaluators of subjective components of study were masked to other aspects of the status of the participants	Describe any assessments undertaken for quality assurance purpose	Explain any patient exclusions from analysis	Describe how confounding was assessed and/or controlled	If applicable, explain how missing data were handled in the analysis	Summarize patient response rates and completeness of data collection	Clarify what follow-up, if any, was expected and the percentage of patient for which incomplete data or follow-up was obtained	Score
Sobczyńska-Malefora et al. 2021 [33]	1	1	1	0	0	1	0	1	1	1	0	7
Guven et al. 2006 [38]	1	1	1	0	0	0	1	1	1	1	0	7
Lai et al. 2018 [40]	1	1	1	1	0	1	0	0	1	1	1	8
Li et al. 2019 [47]	1	1	1	1	0	0	1	1	1	1	0	8
Seghieri et al. 2003 [43]	1	1	1	1	0	0	1	1	0	0	0	6
