# Supplementary figures and images for: The Influence of Maternal Folate Status on Gestational Diabetes Mellitus: A Systematic Review and Meta-Analysis
Source: Nutrients. 2023 Jun 16;15(12):2766. doi: 10.3390/nu15122766 (PMC10300922; doi:10.3390/nu15122766)

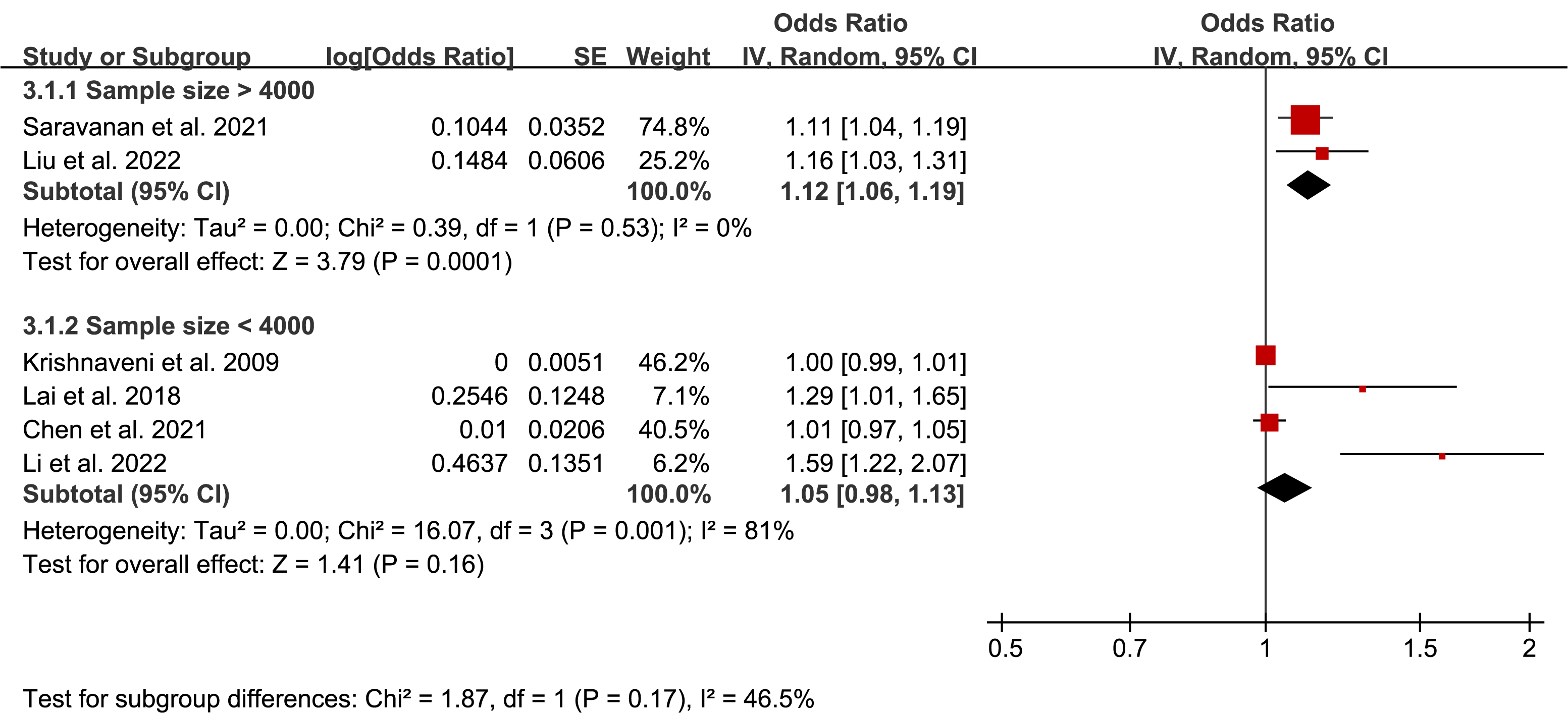

Supplement: Supplementary file 1 [file nutrients-15-02766-s001.zip › Figure S1.tif]

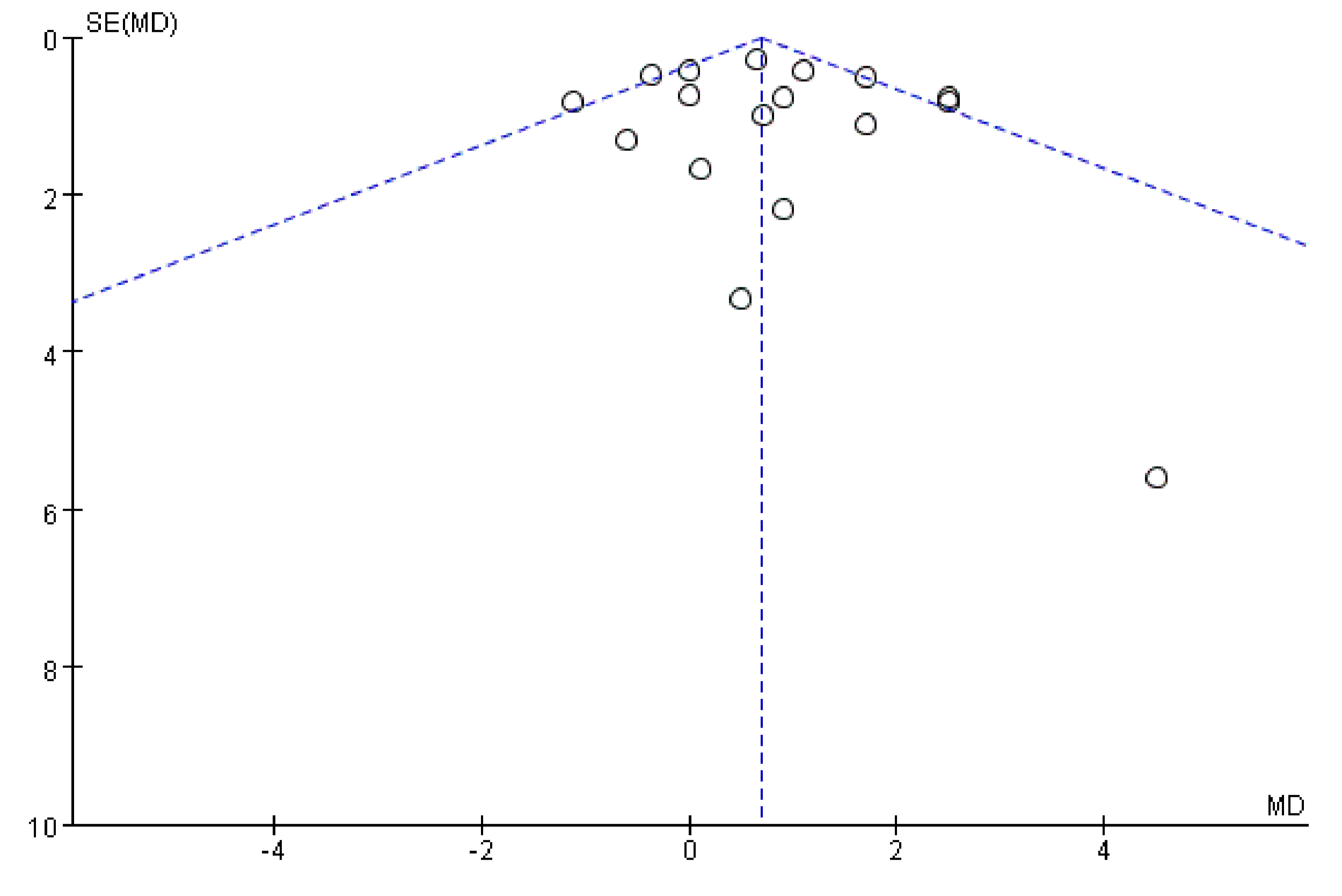

Supplement: Supplementary file 1 [file nutrients-15-02766-s001.zip › Figure S2.tif]
